# Controls on Organic Matter Accumulation of the Triassic Yanchang Formation Lacustrine Shales in the Ordos Basin, North China

**DOI:** 10.1021/acsomega.1c02993

**Published:** 2021-10-01

**Authors:** Xiaoliang Chen, Bin Zhang, Haiping Huang, Zhiguo Mao

**Affiliations:** † School of Energy Resources, China University of Geosciences (Beijing), Beijing 100083, China; ‡ School of Chemistry and Chemical Engineering, Yulin University, Yulin 719000, China; § Research Institute of Petroleum Exploration & Development, PetroChina, Beijing 100083, China; ∥ School of Geosciences, Yangtze University, Wuhan 430100, Hubei, China; ⊥ Department of Geoscience, University of Calgary, Calgary, Alberta T2N 1N4, Canada

## Abstract

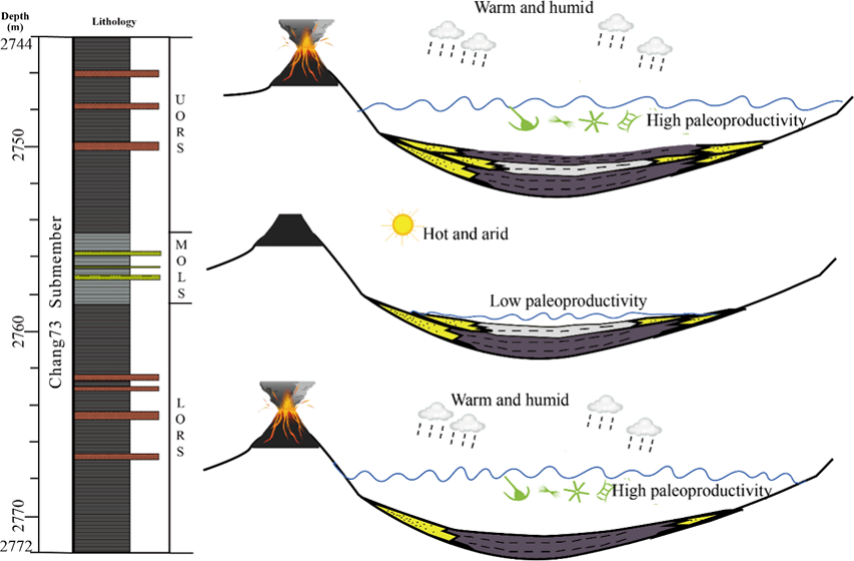

Lacustrine shales in the third submember of the Chang7 (Chang7_3_) of the Triassic Yanchang Formation have the highest oil and gas generation potential in the Ordos Basin, North China. To unravel factors governing organic enrichment within this submember, Rock-Eval pyrolysis, major and trace elemental analyses, and molecular composition of extractable organic matter were applied for redox condition, paleosalinity, dilution effect by terrestrial input, paleoproductivity, and paleoclimate condition investigation. The total organic carbon (TOC) contents of the Chang7_3_ organic-rich lacustrine shales show a tripartite feature and can be divided into the upper organic-rich section (UORS, average TOC 6.8 wt %), the middle organic-lean section (MOLS, average TOC 3.5 wt %), and the lower organic-rich section (LORS, average TOC 6.7 wt %). The variation of the productivity-related paleoclimate is likely the main driving force leading to the change of organic richness within the Chang7_3_ submember. The MOLS was deposited under a relatively hot and arid climate (high Sr/Cu but low Rb/Sr values) with lower paleoproductivity (low *P*_org_/Ti and *P*_org_ values). Additionally, clastic dilution may further reduce the TOC content to a certain extent in the MOLS. The UORS and LORS, however, were deposited under a warm and humid climate, which leads to enhancement of chemical weathering (high Ln(Al_2_O_3_/Na_2_O) values), increased nutrient input, and elevated paleoproductivity. Furthermore, paleoproductivity of UORS and LORS was further boosted by additional key nutrients, such as Fe and P_2_O_5_, provided by syn-depositional volcanic ash. Both paleoredox (U/Th, *C*_org_/*P*, and Pr/Ph) and paleosalinity (Sr/Ba, gammacerane index) proxies suggest no noteworthy variation of redox and salinity conditions throughout the Chang7_3_ interval.

## 1 Introduction

Recently conventional petroleum resources have been partly overshadowed by unconventional oil and gas resources, among which organic-rich shale sourced resources, shale oil and shale gas, have gained great attention for their worldwide distribution not only in marine environments but also in lacustrine successions.^1−4^ The controlling factors of organic matter enrichment in marine environments have been extensively investigated, which can be attributed to the productivity model and the preservation model.^1−3^ In the productivity-driven model, the flux of organic carbon sinking to the seafloor is the leading factor controlling the organic accumulation, while the defining factor of organic matter accumulation in the preservation-driven model is the redox conditions of the water column, with reducing conditions strengthening the preservation of organic matter and oxidizing conditions weakening the preservation of organic matter. Previous studies^4^ had revealed that deposition of lacustrine sediments differs from marine ones not only in scale but also in being more sensitive to changes in climate and clastic supply, resulting in the complexity of organic accumulation in lacustrine systems. Factors including tectonic activity, paleoclimate, redox conditions, paleoproductivity, paleosalinity, paleowater depth, pH value, and others, all exert a certain impact on organic accumulation during the evolution of lacustrine sediments.^5,6^

Lacustrine shales of the Chang7 member with a high total organic carbon (TOC), of up to 30–40%^7^ from the Triassic Yanchang Formation in the Ordos Basin have become attractive targets for shale oil and gas production. Controls on organic matter enrichment in these shales have been investigated by previous studies. Some studies suggested that the strongly reducing bottom-water conditions control the organic matter enrichment of the Chang7 shales,^8,9^ whereas others argued that other factors but not redox condition of the bottom-water play major roles in organic enrichment of the section as the redox condition could be dominant by oxic–suboxic during the Chang7 period.^10,11^ Seawater invasion events governing the concentration of organic matter in the Chang7 member have been proposed,^12^ but no convincing evidence of such an invasion is available.^13^ Paleoclimate as the main control for organic accumulation in the Chang7 shales has been mentioned in the literature,^14^ but no systematic investigation has been carried out.

The Chang7_3_ submember represents a short-term source rock development and organic matter accumulation interval, which has relatively simple constraints. The bulk organic geochemistry, biomarker, and major and trace-element data of samples were thoroughly investigated in the present study. The influence of volcanic ash on syn-depositional black shale was also analyzed. The aim of the present study is to elucidate the major controls on organic matter enrichment in Chang7_3_ shales and to provide a deeper understanding on the formation mechanism of organic-rich shales in the Ordos Basin and other lacustrine basins.

## 2 Geological Setting

**Figure 1:**
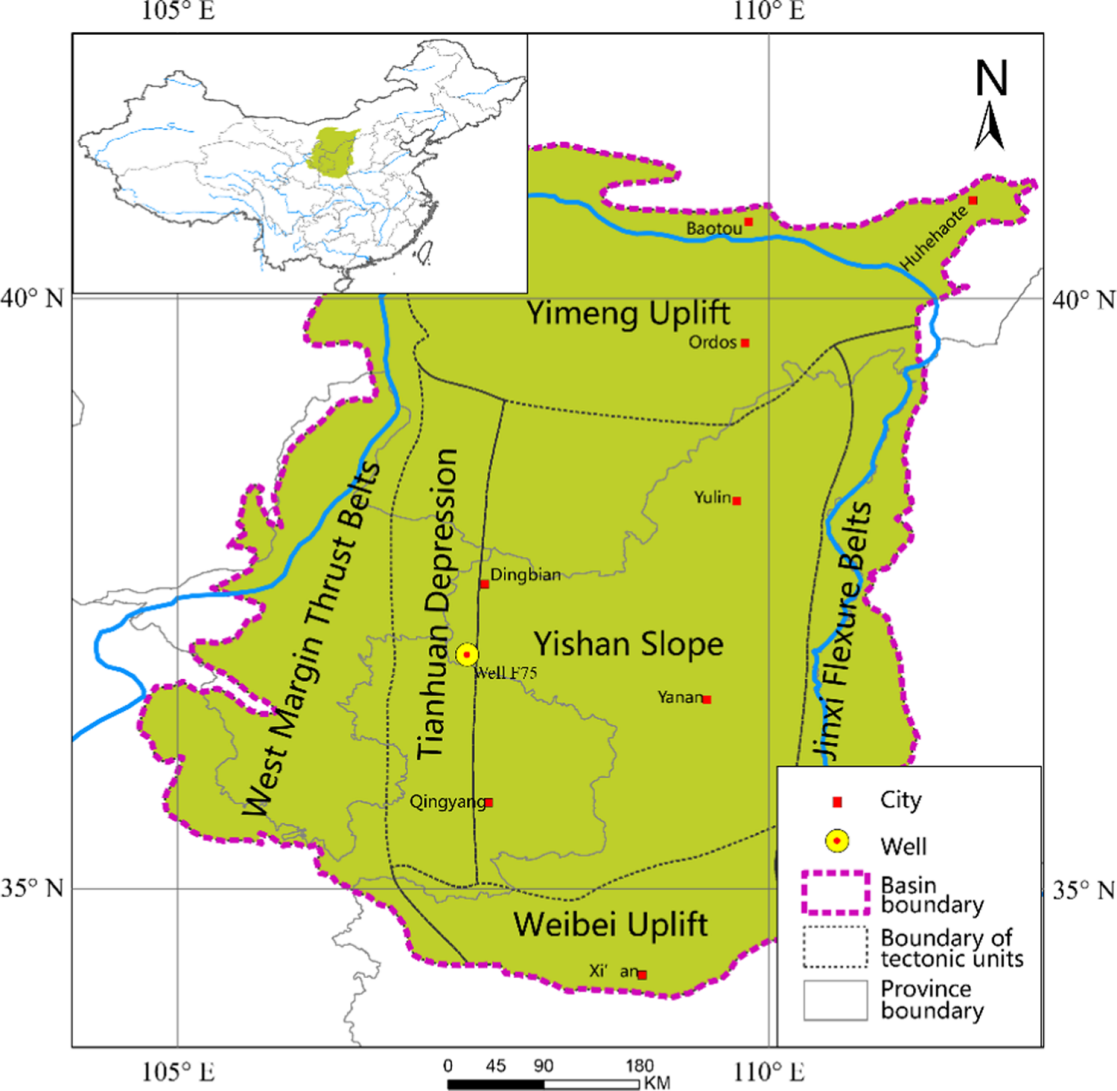
Tectonic frame of the Ordos Basin showing the location of the study well with the inset map showing the basin location in China.

**Figure 2:**
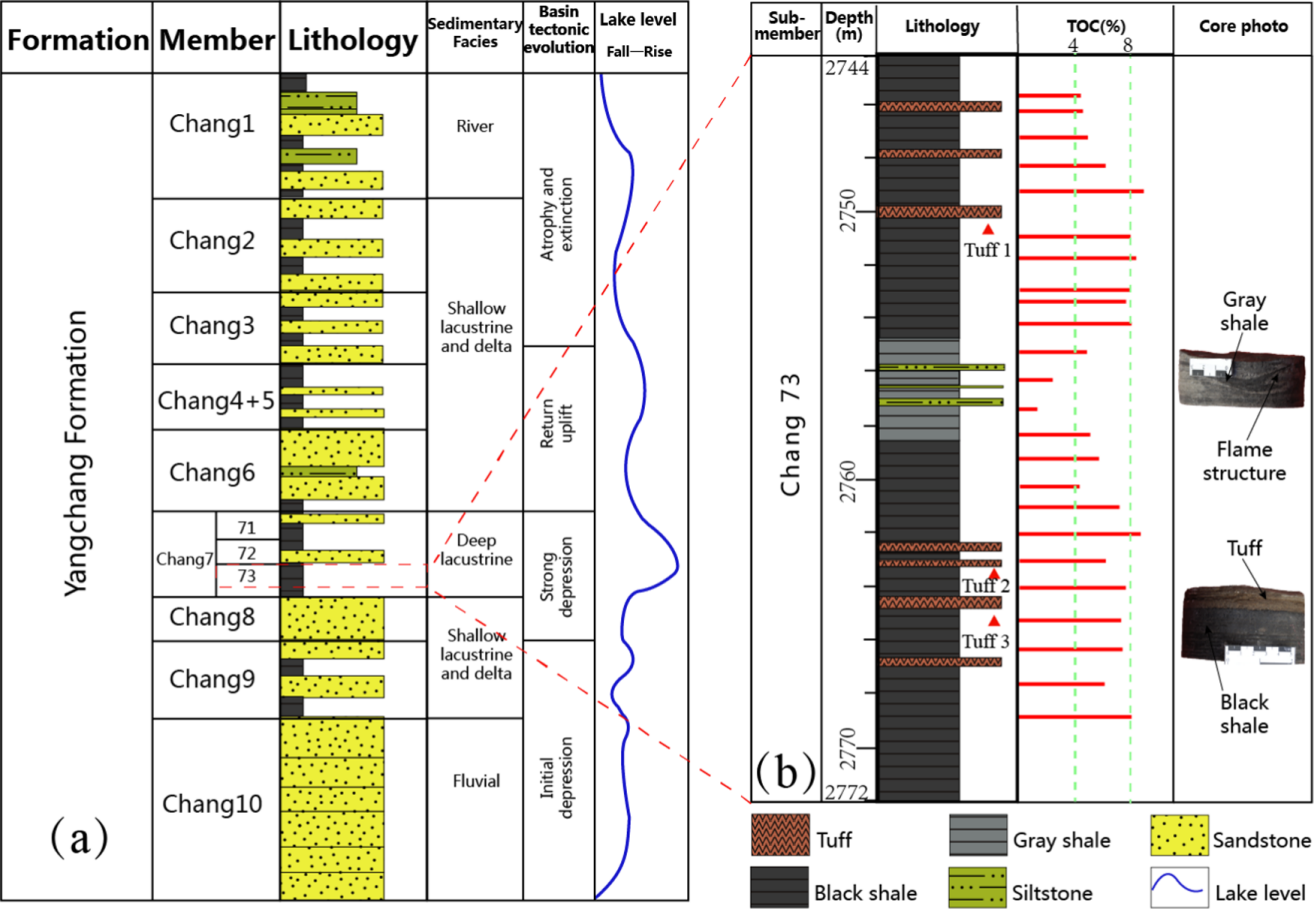
(a) Generalized stratigraphy of the Triassic Yanchang Formation in the Ordos Basin. (b) Stratigraphic column and core photos of the Chang7_3_ submember of Well F75.

The Ordos Basin is an intracratonic basin consisting of six different tectonic units (Yimeng Uplift, West Margin Thrust Belts, Tianhuan Depression, Yishan Slope, Jinxi Flexure Belts, and Weibei Uplift), located in North China.^15^ It underwent a transition from a lacustrine to a fluvial depositional environment in the mid to late Triassic during the Indosinian Orogeny (Figure 1).^16^ The upper Triassic Yanchang Formation bears the most important source rocks in the whole basin, which can be divided into 10 members (Chang1 to Chang10 members from the top to bottom) on the basis of the occurrence of oil pay zones, marker beds, and sequence stratigraphic cycles (Figure 2a).^17^ Widely distributed black organic-rich shales corresponding to the maximum transgression system tract across the basin feature prominently in the Chang7 member.^16^ The Chang7 member comprises three submembers from the top to bottom, namely, Chang7_1_, Chang7_2_, and Chang7_3_, among which Chang7_3_ is characterized by thick black shales interbedded with multiple tuff layers.^18^ Ancient volcanos located near the southwest edge of the basin may be responsible for the formation of these tuff layers.^19^

## 3 Samples and Methods

To closely investigate the vertical organic heterogeneity of the Chang7_3_ submember, a suit of 23 core samples was collected along 30 m thick black shales (sampling space around 1 meter apart) in Well F75 from the Chang7_3_ submember in the Tianhuan Depression, southwest of the Ordos Basin (Figure 2b). Three tuff samples within the submember were also collected (Figure 2) for major element analysis.

A Leco CS–230 elemental analyzer was used to measure the TOC of all 23 core samples within which carbonate minerals were removed using dilute HCl before the TOC measurement. About 50 mg of unextracted powder (100 mesh) of each core was taken to conduct the pyrolysis analysis using the Rock-Eval VI apparatus. The flame ionization detector equipped on the apparatus detects the hydrocarbons and CO_2_ released under the standard pyrolysis program. For details of Rock-Eval VI pyrolysis procedures and parameters, refer to Behar.^20^

After cleaning and dryness, powdered samples (less than 200 mesh) were analyzed with AB104L Axios mAx X-ray fluorescence (XRF) spectrometry to determine concentrations of major elements. The GB/T 14506.14–2010 standard was used as the reference. The XRF analytical accuracy was within 5% error bar. Trace elements were analyzed using a Finnigan inductively coupled plasma mass spectrometer (ICP-MS) under the conditions of 23 °C and 26% humidity, using GB/T 14506.30-2010 as the standard. The measurement precision was within 0.1% error bar.

Extricable organic matter (EOM) was obtained from crushed core samples (80–100 mesh) by Soxhlet extraction with dichloromethane for 72 h. The asphaltene within the EOM was removed by adding excessive cold *n*-heptane into the extract for precipitation. The maltene fraction was then separated into saturated hydrocarbons, aromatic hydrocarbons, and resins eluted by hexane, toluene, and dichloromethane sequentially on a liquid chromatography column. Known amounts of internal standards (5-α androstane, *n*-tetracosane-d50, and anthracene-d10) were added into the saturated and aromatic hydrocarbon fractions for quantitative purposes. Then, the saturated hydrocarbon fraction was analyzed with a Thermo-Trace GC Ultra-DSQ II gas chromatography–mass spectrometry (GC–MS) system equipped with an HP-5MS (60 m × 0.25 mm × 0.25 μm) elastic quartz capillary column. The initial temperature of the oven was programmed from 40 °C for 5 min, then increased to 320 °C at 4 °C/min, and held for 20 min with helium as the carrier gas (constant flow rate of 1 mL/min). Data was obtained in the selected ion monitoring (SIM) model with an electron energy of 70 eV. The qualification and quantification analyses of biomarkers were carried out using the software XCALIBUR. Peak area was used for quantitation, and no response factor calibration was performed.

Except for the major and trace-element analyses, which were measured at the Analytical Laboratory of the Beijing Research Institute of Uranium Geology, all of the other experiments were performed at the Petroleum Geology Research and Laboratory Center, RIPED.

## 4 Results

### 4.1 Bulk Organic Geochemistry

**Figure 3:**
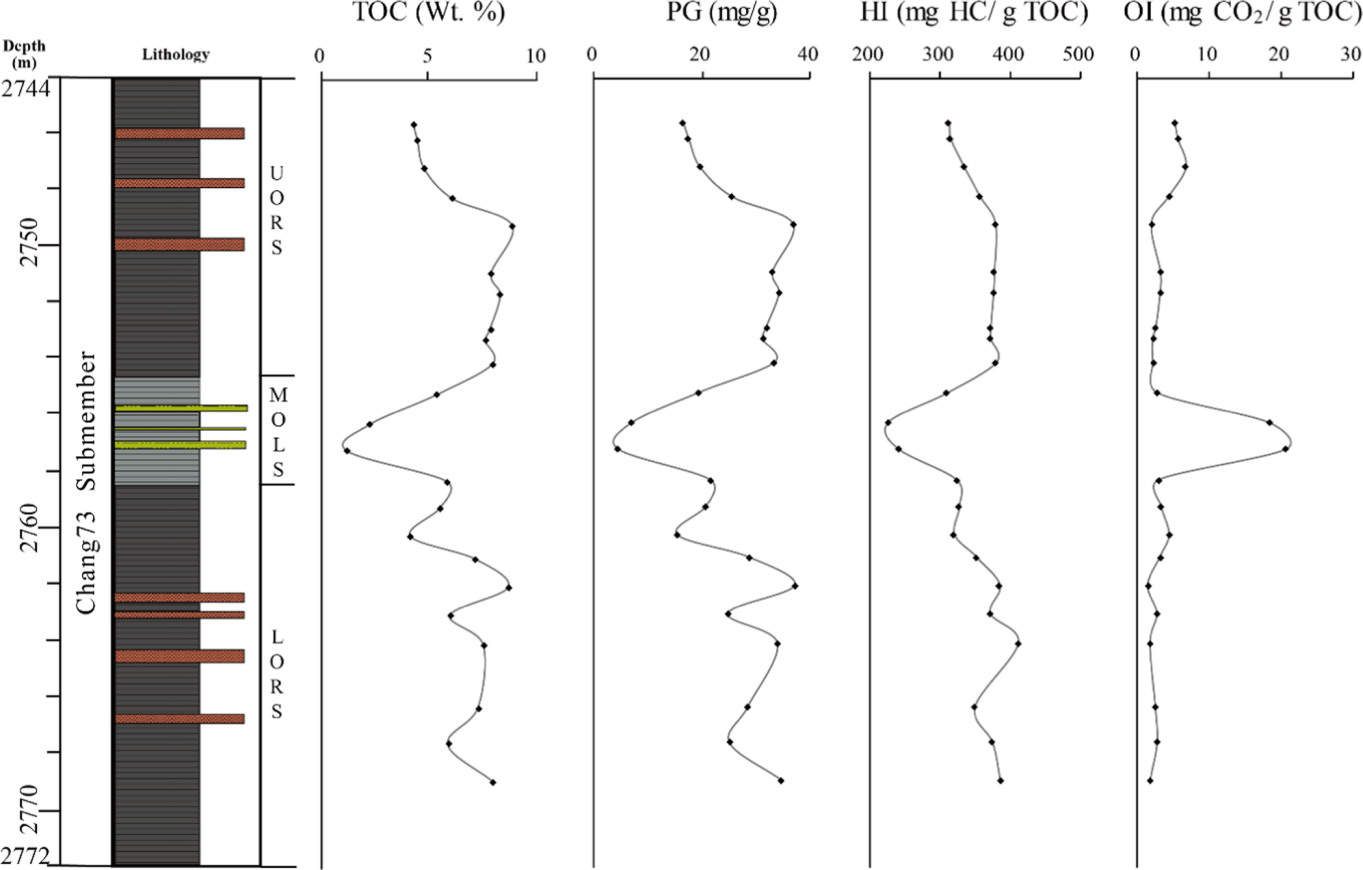
Bulk geochemistry characteristics of the Chang7_3_ submember shale in Well F75 of the Ordos Basin. For average values of TOC, generation potential (PG), hydrogen index (HI), and oxygen index (OI), please refer to Table 1.

Bulk geochemistry characteristics of the samples are listed in Table 1. The TOC contents range from 1.2 to 8.8 wt % (average 5.8 wt %), suggesting that the Chang7_3_ black shales are good to excellent source rocks. Vertically, the upper and lower sections of the Chang7_3_ submember show a higher TOC content (4.2–8.8 wt %, average 6.8 and 4.7–8.6 wt %, average 6.7 wt %, respectively) than that in the middle section (1.2–5.7 wt %, average 3.5 wt %) (Figure 2), which can be further divided into the upper organic-rich section (UORS), the middle organic-lean section (MOLS), and the lower organic-rich section (LORS) (Table 1 and Figure 3).

**Table 1:** Bulk Geochemical Parameters of Samples from the Chang7_3_ Submember of Well F75^a^

sample ID	depth (m)	lithology	TOC (wt %)	*S*_1_ (mg/g)	*S*_2_ (mg/g)	PG (mg/g)	HI	OI	*T*_max_ (°C)	Roc (%)
1	2745.57	Black Shale	4.2	3.18	13.20	16.38	311.5	5.2	440	0.76
2	2746.17	Black Shale	4.4	3.52	13.92	17.44	313.8	5.6	440	0.76
3	2747.17	Black Shale	4.7	3.70	15.81	19.51	332.9	6.7	438	0.72
4	2748.22	Black Shale	6.0	4.00	21.50	25.50	355.8	4.3	444	0.83
5	2749.17	Black Shale	8.8	3.50	33.20	36.70	377.4	1.9	448	0.90
6	2750.87	Black Shale	7.8	3.52	29.49	33.01	376.1	3.2	448	0.90
7	2751.65	Black Shale	8.3	3.20	30.92	34.12	374.7	3.2	447	0.89
8	2752.84	Black Shale	7.8	2.91	28.88	31.79	370.8	2.6	445	0.85
9	2753.27	Black Shale	7.6	3.05	28.10	31.15	371.0	2.1	446	0.87
10	2754.10	Black Shale	7.9	3.39	29.90	33.29	378.7	2.2	447	0.89
11	2755.18	Gray Shale	5.3	2.62	16.48	19.10	308.9	2.8	444	0.83
12	2756.24	Gray Shale	2.2	1.82	5.03	6.85	224.3	18.3	436	0.69
13	2757.17	Gray Shale	1.2	1.48	2.78	4.26	239.2	20.6	441	0.78
14	2758.26	Gray Shale	5.8	2.77	18.62	21.39	321.7	2.9	447	0.89
15	2759.19	Black Shale	5.5	2.77	17.87	20.64	326.1	3.3	446	0.87
16	2760.20	Black Shale	4.1	2.15	13.10	15.25	319.0	4.4	438	0.72
17	2761.00	Black Shale	7.1	3.81	24.77	28.58	350.1	3.1	447	0.89
18	2762.00	Black Shale	8.6	3.93	33.12	37.05	383.8	1.4	447	0.89
19	2763.00	Black Shale	6.0	2.62	22.05	24.67	370.2	2.7	447	0.89
20	2764.05	Black Shale	7.5	3.26	30.60	33.86	409.7	1.7	447	0.89
21	2766.30	Black Shale	7.2	3.23	25.16	28.39	348.5	2.5	447	0.89
22	2767.58	Black Shale	5.8	3.23	21.76	24.99	373.2	2.7	445	0.85
23	2768.90	Black Shale	7.9	4.14	30.40	34.54	384.2	1.8	447	0.89

a TOC, total organic carbon; *S*_1_, free hydrocarbon; *S*_2_, remaining hydrocarbon potential; PG, generation potential = *S*_1_ + *S*_2_; HI, hydrogen index = *S*_2_/TOC × 100; OI, oxygen index = *S*_3_/TOC × 100; *T*_max_, peak pyrolysis temperature; Roc, calculated equivalent vitrinite reflectance = 0.018 × *T*_max_ – 7.16.

**Figure 4:**
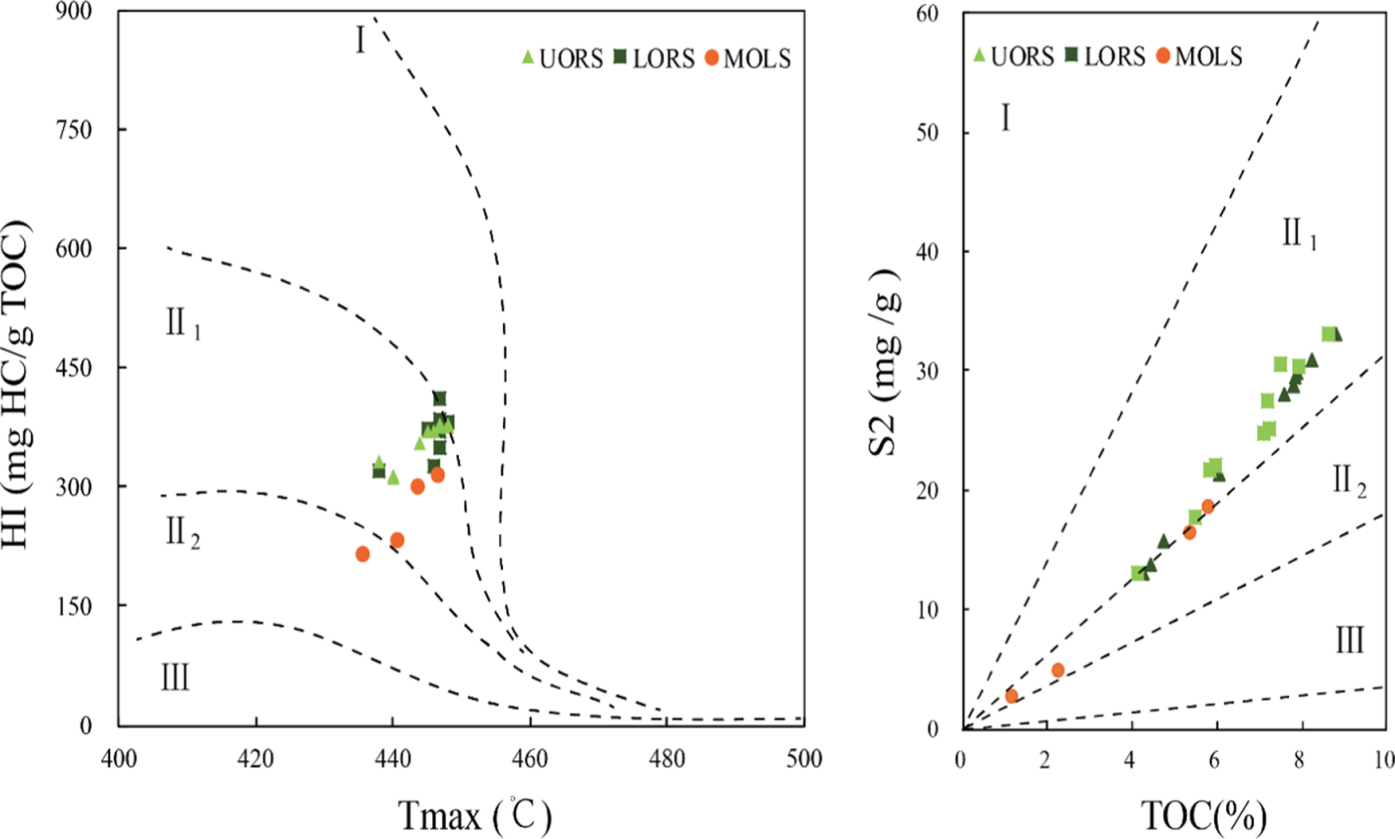
Cross-plots of HI versus *T*_max_ and S2 versus TOC of samples from the Chang7_3_ submember in Well F75 of the Ordos Basin.

The hydrocarbon generation potential (PG = S1 + S2) and the hydrogen index (HI = S2/TOC × 100) show the same vertical variation trend as TOC contents. The PG values vary from 16.4 to 36.7 mg/g rock (average 27.9 mg/g rock) and from 15.3 to 37.1 mg/g rock (average 28.0 mg/g rock) in the UORS and LORS, respectively. Lower PG values ranging from 4.3 to 21.4 mg/g rock (average 12.9 mg/g rock) occur in the MOLS. The HI values vary from 311.5 to 378.7 mg HC/g TOC (average 356.3 mg HC/g TOC) and from 319.0 to 409.7 mg HC/g TOC (average 364.7 mg HC/g TOC) in UORS and LORS, respectively, while those in the MOLS range from 224.3 to 321.7 mg HC/g TOC (average 273.5 mg HC/g TOC). The oxygen index (OI = S3/TOC × 100) values in the MOLS are higher than those in the UORS and the LORS, showing the opposite trend to the TOC (Figure 3). The *T*_max_ values in the UORS and LORS are mostly in the range of 440–448 °C, while a slightly wide range of variation, mostly from 436 to 447 °C, occurs in the MOLS (Table 1 and Figure 4).

### 4.2 Biomarkers

**Figure 5:**
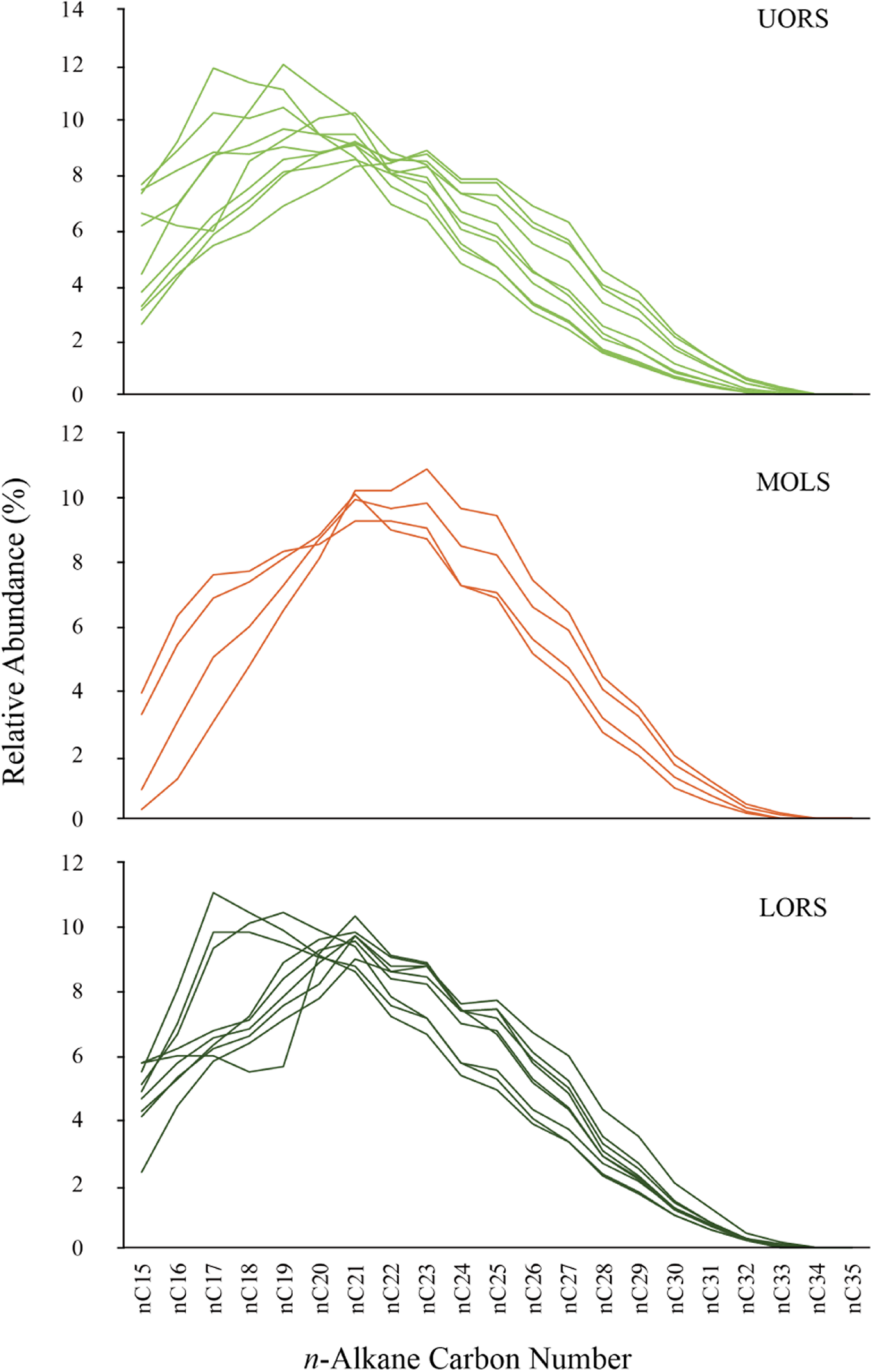
Distribution of the relative abundance of *n*-alkanes in the studied samples from the Chang7_3_ submember of the Ordos Basin.

*n*-Alkanes (ranging from *n*-C_15_ up to *n*-C_35_) are the most abundant compound class in EOM and can be easily identified from mass chromatograms (*m*/*z* 85). While the relative abundance of the *n*-alkane components in all samples shows a unimodal distribution pattern, the highest abundant component varies in three studied sections. The *n*-alkanes are mainly maximized at *n*-C_17_ and *n*-C_19_ in both UORS and LORS sections, whereas the maximized carbon peak occurs at *n*-C_21_ or *n*-C_23_ in MOLS (Figure 5 ).

All samples show relatively low pristane (Pr)-to-phytane (Ph) ratios (0.5–1.0, average 0.8). The ratios of Pr/*n*-C_17_ are in the range of 0.12–0.20, 0.13–0.15, and 0.15–0.23 and the Ph/*n*-C_18_ ratios are 0.16–0.26, 0.16–0.17, and 0.17–0.24 for the UORS, MOLS, and LORS, respectively. A slightly odd-over-even carbon number preference can be observed, with carbon preference index (CPI)^21^ values ranging from 1.12 to 1.18 without obvious variation in sample sections.

**Figure 6:**
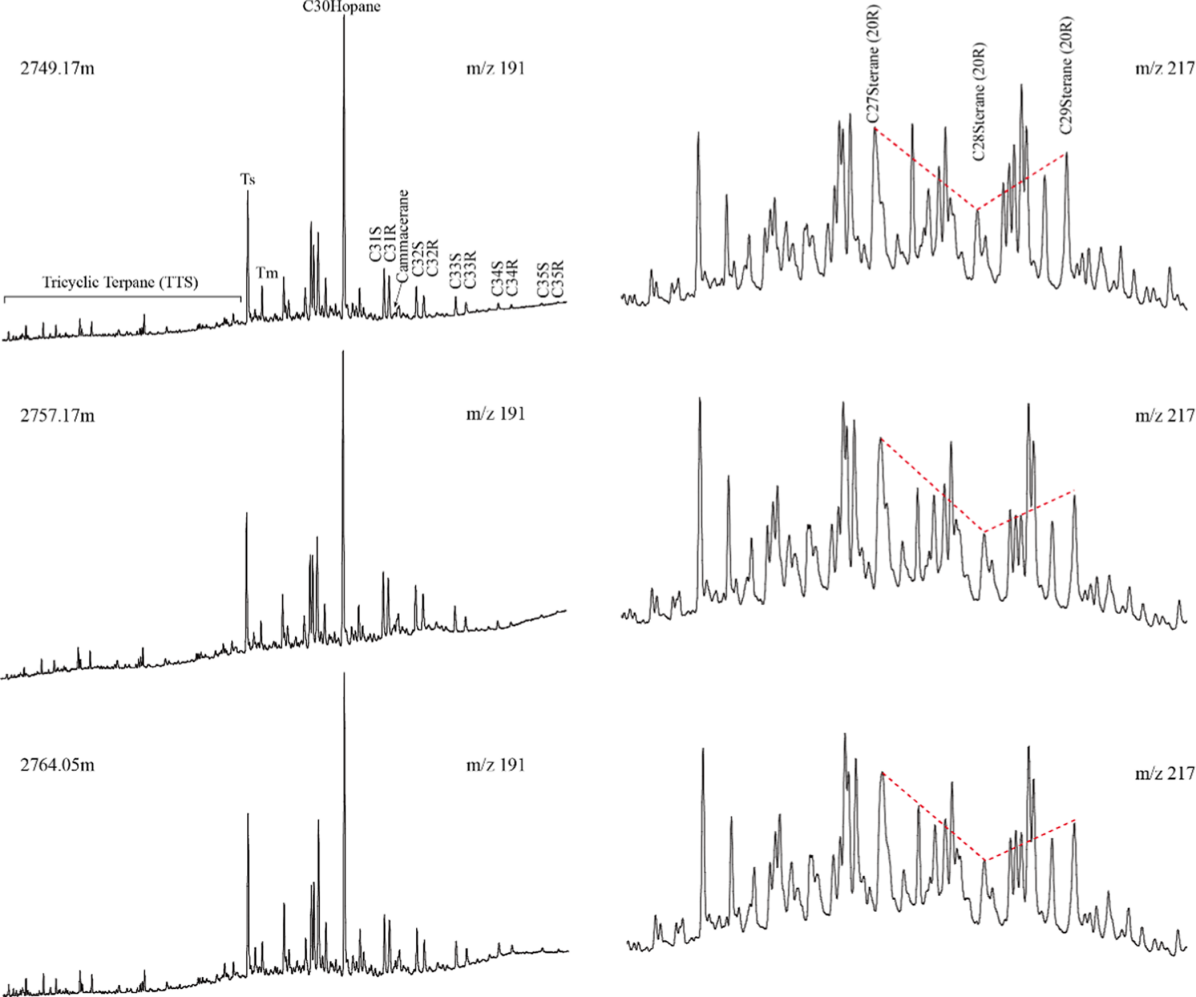
Representative *m*/*z* 191 (left) and *m*/*z* 217 (right) mass chromatograms showing the distribution of terpanes and steranes in samples from the Chang7_3_ submember of Well F75 of the Ordos Basin.

The terpanes in the *m*/*z* 191 mass chromatograms show the predominance of pentacyclic terpenes (PTs) over tricyclic terpanes (TTs) with 17α,21β-C_30_ hopane (C_30_H) as the dominated peak followed by 18α-22,29,30-C_27_ trisnorneohopane (*T*_s_) and 17α,21β-C_29_ hopane (C_29_H) (Figure 6 left). The gammacerane index (GI = gammacerane/C_30_H) is relatively low and varies slightly across the whole Chang7_3_ submember (0.01–0.06) (Table 2). The *T*_s_/(*T*_s_ + *T*_m_) (*T*_m_, 17α(H)-22,29,30-trisnorhopane) and C_29_*T*_s_/(C_29_*T*_s_ + C_29_H) ratios are in the range of 0.80–0.84 and 0.42–0.51, respectively (Table 2). The relative abundance of homohopanes decreases sharply with increasing carbon number, and C_35_ homohopanes are absent in most samples (Figure 6 left).

**Table 2:** Biomarker Parameters of Samples from the Chang7_3_ Submember in Well F75^a^

sample ID	depth (m)	CPI	TAR	Pr/*n*-*C*_17_	Ph/*n*- *C*_18_	Pr/Ph	*T*_s_/(*T*_s_ + *T*_m_)	22S/(22S + 22) *C*_31_H	ββ/(αα + ββ) *C*_29_ sterane	20S/(20S + 20R) *C*_29_ sterane	GI	*C*_27_ sterane (%)	*C*_28_ sterane (%)	*C*_29_ sterane (%)
1	2745.57	1.12	0.74	0.19	0.25	0.7	0.84	0.49	0.55	0.42	0.03	35	30	36
2	2746.17	1.15	0.46	0.20	0.26	0.7	0.84	0.50	0.52	0.40	0.02	40	28	33
3	2747.17	1.14	0.59	0.20	0.25	0.7	0.82	0.50	0.56	0.40	0.02	38	27	35
4	2748.22	1.15	0.59	0.19	0.24	0.7	0.81	0.50	0.54	0.42	0.03	40	27	34
5	2749.17	1.17	0.15	0.17	0.19	0.9	0.80	0.51	0.54	0.43	0.03	37	26	37
6	2750.87	1.16	0.28	0.19	0.22	0.8	0.80	0.51	0.55	0.39	0.04	38	28	34
7	2751.65	1.18	0.15	0.14	0.16	0.7	0.81	0.51	0.55	0.40	0.02	40	28	32
8	2752.84	1.18	0.21	0.15	0.17	0.9	0.80	0.51	0.55	0.43	0.02	35	29	37
9	2753.27	1.18	0.26	0.12	0.17	0.5	0.81	0.52	0.55	0.38	0.01	38	29	32
10	2754.10	1.17	0.12	0.16	0.22	0.8	0.81	0.52	0.55	0.41	0.02	37	30	33
11	2755.18	1.18	0.34	0.14	0.16	0.9	0.82	0.52	0.56	0.41	0.02	40	30	30
12	2756.24	1.16	0.76	0.13	0.16	0.7	0.83	0.51	0.55	0.41	0.04	39	30	31
13	2757.17	1.16	1.13	0.14	0.16	0.5	0.84	0.51	0.56	0.39	0.04	39	30	32
14	2758.26	1.14	0.42	0.15	0.17	0.8	0.83	0.51	0.55	0.35	0.03	41	27	32
15	2759.19	1.14	0.37	0.15	0.17	0.8	0.83	0.50	0.56	0.37	0.06	39	28	34
16	2760.20	1.13	0.70	0.18	0.20	0.8	0.83	0.49	0.57	0.38	0.04	38	29	32
17	2761.00	1.13	0.47	0.15	0.17	1.0	0.83	0.50	0.53	0.46	0.03	39	30	31
18	2762.00	1.15	0.34	0.16	0.18	0.9	0.83	0.50	0.56	0.42	0.04	42	27	31
19	2763.00	1.17	0.41	0.17	0.18	0.9	0.84	0.50	0.55	0.39	0.03	41	29	31
20	2764.05	1.14	0.21	0.23	0.24	1.0	0.84	0.49	0.55	0.39	0.04	38	30	32
21	2766.30	1.14	0.48	0.17	0.18	0.9	0.84	0.51	0.56	0.38	0.03	40	30	30
22	2767.58	1.14	0.27	0.21	0.24	0.9	0.82	0.50	0.55	0.39	0.04	41	29	31
23	2768.90	1.14	0.22	0.18	0.21	0.8	0.84	0.50	0.54	0.40	0.04	37	31	32

a CPI, carbon preference index = (*C*_13_ + *C*_15_ + *C*_17_ + *C*_19_ + *C*_21_ + *C*_23_ + *C*_25_ + *C*_27_ + *C*_29_ + *C*_31_)/(*C*_12_ + *C*_14_ + *C*_16_ + *C*_18_ + *C*_20_ + *C*_22_ + *C*_24_ + *C*_26_ + *C*_28_ + *C*_30_) for *n*-alkanes; TAR, terrigenous: aquatic ratio = (*C*_27_ + *C*_29_ + *C*_31_)/(*C*_15_ + *C*_17_ + *C*_19_) for *n*-alkanes; GI, gammacerane index = gammacerane/C_30_H.

Steranes detected in *m*/*z* 217 mass chromatograms show a similar abundance of diasteranes and regular steranes. Most samples have C_27_ steranes more abundant than their C_28_ and C_29_ counterparts and show an “L”-shaped distribution pattern, while some samples have equally abundant C_27_ and C_29_ steranes but much lower C_28_ steranes and show a “V”-shaped distribution pattern (Figure 6 right). For instance, the averaged relative abundances of C_27_, C_28_, and C_29_ steranes are 38.7, 28.7, and 32.7% in the UORS, respectively (Table 2). The C_29_ sterane ββ/(ββ + αα) ratios are in the range of 0.52–0.57, suggesting that the Chang7_3_ submember falls in the oil-generation window.

### 4.3 Elemental Geochemistry

Partial major and trace-element contents of shale samples are shown in Table 3. Among the major elements, Al_2_O_3_ shows high concentrations across the whole section (15.69–21.18 wt %, average 17.77 wt %), while P_2_O_5_ presents low and variable concentrations in different sections with 0.27–0.66 wt % (average 0.42 wt %) in the UORS, 0.28–0.41 wt % (average 0.35 wt %) in the MOLS, and 0.27–0.82 wt % (average 0.41 wt %) in the LORS. Compared to shale samples, tuff samples contain a much lower abundance of P_2_O_5_ and Fe (0.06–0.11 and 1.37–1.68 wt %, respectively) (Table 4). Concentrations of Ti show a relatively constant value throughout the Chang7_3_ submember (average 0.75 wt %). Ratios of specific trace elements such as Rb/Sr and Sr/Cu vary substantially in different sections. Rb/Sr ratios in the UORS and LORS (average 0.56 and 0.60, respectively) are higher than those in the MOLS (average 0.36), whereas Sr/Cu ratios show an opposite trend with higher values (average 3.74) in the MOLS than those in the UORS (average 2.62) and the LORS (average 3.22), respectively. Most samples have their Sr/Ba ratios below 0.6 except for a few samples from LORS with Sr/Ba ratios in the range of 0.6–1.0 (Table 3).

**Table 3:** Concentrations of Major and Trace Elements and Some Relevant Inorganic Geochemical Proxies of Samples from the Chang7_3_ Submember in Well F75

	major elements (%)								trace elements (ppm)					parameters								
depth (m)	A1_2_O_3_	SiO_2_	Fe	CaO	Na_2_O	K_2_O	P_2_O_5_	Ti_2_O	Mo	Ba	Cu	Rb	Sr	*P*_org_ (%)	*P*_org_/Ti	Ln(Al_2_O_3_/Na_2_O)	Si/Al	Sr/Ba	Rb/sr	Sr/Cu	U/Th	*C*_org_/*P* (mol ratio)
2745.57	19.64	57.70	4.46	0.54	0.85	2.92	0.27	0.88	4	573	98.4	158	184	0.10	0.11	2.65	2.94	0.32	0.86	1.87	0.38	41.00
2746.17	17.07	57.57	6.70	0.70	0.90	2.64	0.36	0.79	10	545	94.5	141	192	0.21	0.26	2.45	3.37	0.35	0.73	2.03	0.26	32.01
2747.17	17.71	56.56	5.77	0.76	0.83	2.94	0.36	0.79	5	557	52.6	153	200	0.20	0.26	2.56	3.19	0.36	0.77	3.80	0 38	34.18
2748.22	16.22	56 61	6.24	0.89	0.87	2.60	0.42	0.77	9	552	101	136	208	0.28	0.36	2.42	3.49	0.38	0.65	2.06	0.38	37.07
2749.17	17.12	52.90	6.77	0.68	0.81	2.61	0.39	0.75	10	593	110	131	247	0.24	0.32	2.56	3.09	0.42	0.53	2.25	0.36	58.73
2750.87	19.14	52.31	5.08	0.83	0.83	2.71	0.57	0.70	15	761	138	144	372	0.40	0.57	2.64	2.73	0.49	0.39	2.70	0.29	35.85
2751.65	18.12	52.50	5.74	0.86	0.80	2.74	0.44	0.70	11	634	105	158	273	0.28	0.40	2.62	2.90	0.43	0.58	2.60	0.30	48.45
2752.84	16.03	46.95	10.84	1.85	0.61	2.38	0.45	0.69	36	603	113	122	333	0.31	0.45	2.77	2.93	0.55	0.37	2.95	0.33	44.71
2753.27	17.31	51.76	6.80	1.24	0.77	2.63	0.66	0.67	16	812	131	128	480	0.50	0.75	2.62	2.99	0.59	0.27	3.66	0.63	29.87
2754.10	17.83	53.65	5.81	0.62	0.69	2.67	0.32	0.73	16	621	144	141	323	0.17	0.23	2.75	3.01	0.52	0.44	2.24	0.39	63.14
2755.18	17.09	59.48	4.57	0.74	1.22	2.43	0.33	0.65	9	627	118	112	291	0.19	0.29	2.14	3.48	0.46	0.38	2.47	0.48	41.26
2756.24	17.57	54.39	6.89	2.15	1.25	2.19	0.41	0.89	2	884	64.5	99.3	310	0.26	0.29	2.15	3.10	0.35	0.32	4.81	0.53	14.00
2757.17	21.18	58.70	3.33	1.06	1.37	2.62	0.36	0.97	2	920	104	112	382	0.18	0.18	2.24	2.77	0.42	0.29	3.67	0.68	8.32
2758.26	19.74	54.85	5.46	0.55	0.68	2.56	0.28	0.79	6	688	89.8	154	360	0.11	0.14	2.87	2.78	0.52	0.43	4.01	0.80	52.65
2759.19	19.76	55.97	4.47	0.52	0.64	2.63	0.32	0.80	4	604	71.8	139	373	0.15	0.19	2.94	2.83	0.62	0.37	5.19	0.80	43.97
2760.20	18.17	55.44	6.22	1.52	0.42	2.84	0.31	0.78	6	518	117	201	264	0.15	0.19	3.27	3.05	0.51	0.76	2.26	0.87	34.67
2761.00	16.17	54.62	5.73	1.59	0.65	2.53	0.52	0.71	8	651	116	162	366	0.38	0.54	2.71	3.38	0.56	0.44	3.16	0.27	34.88
2762.00	17.00	52.76	5.57	1.10	0.66	2.57	0.38	0.72	8	700	131	172	341	0.24	0.33	2.75	3.10	0.49	0.50	2.60	0.47	58.05
2763.00	15.69	54.32	5.65	3.28	0.53	2.13	0.82	0.54	6	702	54	129	546	0.69	1.27	2.89	3.46	0.78	0.54	2.48	0.34	18.70
2764.05	17.36	53.22	6.04	0.95	0.63	2.71	0.39	0.66	12	676	139	187	327	0.24	0.36	2.81	3.07	0.48	0.57	2.35	0.36	49.85
2766.30	16.96	53.30	6.23	1.32	0.51	2.71	0.39	0.70	9	516	99.8	175	290	0.24	0.35	3.01	3.14	0.56	0.60	2.91	0.46	47.69
2767.58	17.52	55.76	5.44	1.09	0.50	2.67	0.27	0.77	5	504	53.1	176	248	0.12	0.15	3.05	3.18	0.49	0.71	4.67	1.03	55.99
2768.90	18.24	53.57	5.40	0.74	0.42	2.79	0.30	0.77	6	500	65	189	216	0.14	0.18	3.29	2.94	0.43	0.88	3.35	1.40	69.05

**Table 4:** Contrast of Major Element Contents among Chang7_3_ Shale, Chang7_3_ Tuff, and Fresh Volcanic Dust

lithology	Al_2_O_3_ (%)	SiO_2_ (%)	CaO (%)	Na_2_O (%)	K_2_O (%)	Ti_2_O (%)	P_2_O_5_ (%)	Fe (%)
Chang7_3_ tuff 1	27.31	55.08	0.48	1.25	2.14	0.17	0.06	1.68
Chang7_3_ tuff 2	29.26	51.93	0.97	1.35	2.01	0.17	0.08	1.49
Chang7_3_ tuff 3	20.97	64.48	0.41	1.00	1.69	0.16	0.11	1.37
Chang7_3_ tuff average	25.85	57.16	0.62	1.20	1.95	0.17	0.08	1.51
Chang7_3_ shale average	17.77	54.56	1.11	0.76	2.62	0.75	0.41	5.88
fresh volcanic dust average	16.37	58.37	5.59	4.17	1.63	0.80	0.27	4.90

## 5 Discussion

### 5.1 Organic Matter Type and Maturity

Plots of *T*_max_ versus HI and S2 versus TOC both indicate that kerogen types within UORS and LORS are mainly type II_1_, whereas the kerogen types in MOLS are mainly composed of types II_1_ and II_2_ (Figure 4). The main sources of organic matter within UORS, LORS, and MOLS are aquatic planktons with small amounts of terrestrial plants, whereas MOLS contains relatively higher amounts of terrestrial plants. TAR (terrigenous-to-aquatic ratio) is a commonly used parameter to demonstrate terrestrial or aquatic organic matter inputs of source rocks.^22^ TAR values (Table 2 and Figure 10b) further support the organic matter source characteristics within the Chang7_3_ submember.

**Figure 7:**
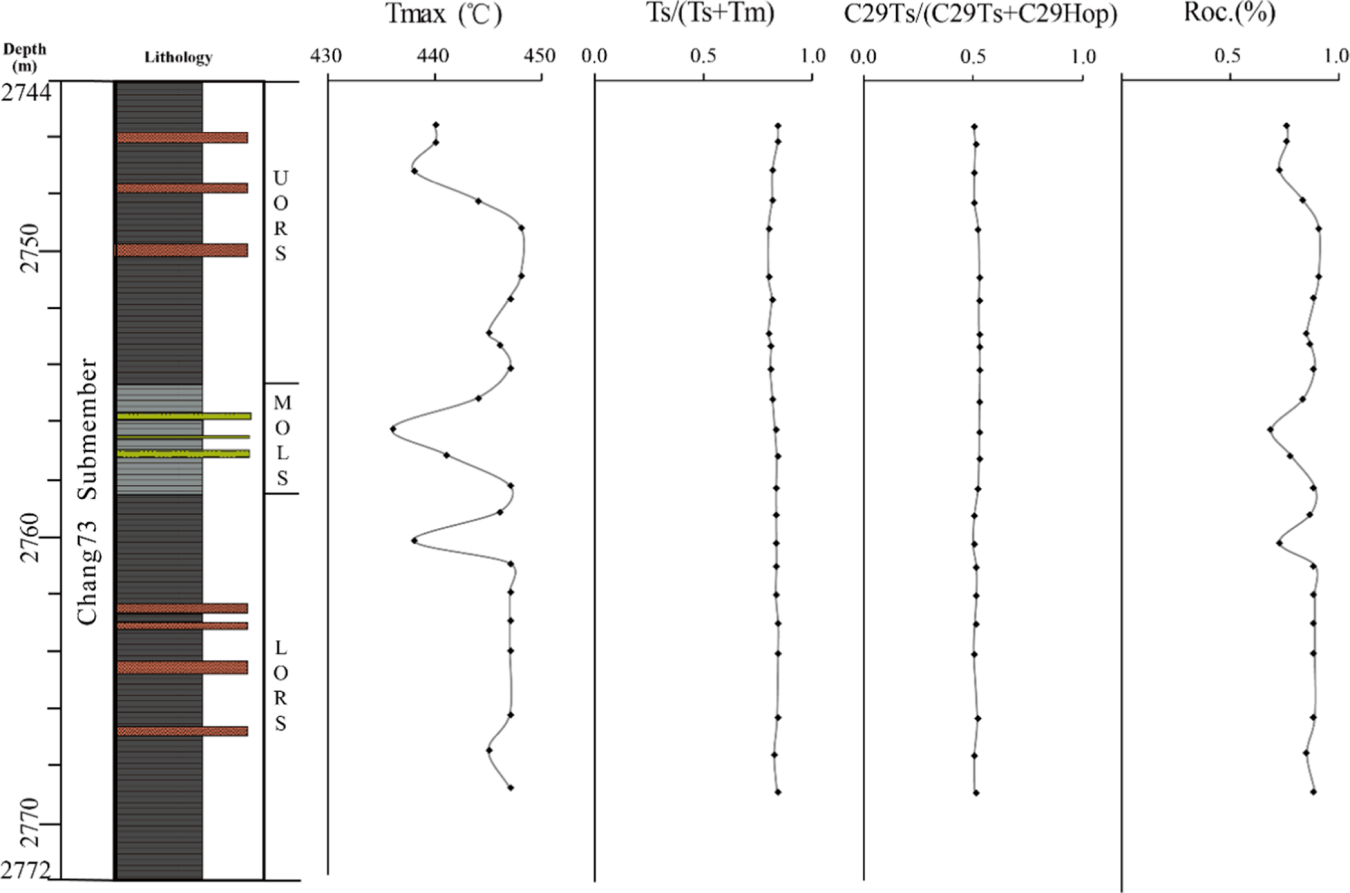
Profiles of maturity parameters within the Chang7_3_ submember of Well F75.

Biomarker maturity parameters, such as *T*_s_/(*T*_s_ + *T*_m_) and C_29_*T*_s_/(C_29_*T*_s_ + C_29_H), which can indicate a much wider range of maturity variation for samples with similar organofacies and approaches the equilibrium point around 1.0 at *R*_o_ of 1.1%,^23^ were used to indicate the maturity levels of present shale samples. All of these ratios indicate the Chang7_3_ shale samples have reached a high maturity level near the peak oil-generation stage (Figure 7), which was further supported by the vitrinite reflectance (*R*_o_, 0.82–0.95%) data of the Chang7 member measured at adjacent wells.^24^ Additionally, the Rock-Eval *T*_max_ values of all samples fall in the oil-generation range with the calculated equivalent vitrinite reflectance (Roc, proposed by Jarvie^25^) >0.7% (Table 1, average 0.84%). Slightly lower *T*_max_ values (Table 1 and Figure 7) in samples from MOLS than those in the UORS and LORS are likely caused by the variation of organofacies and kerogen types. Compared with its original TOC, residual TOC is expected to gradually increase from low maturity to high maturity of the same kerogen type due to hydrocarbon generation.^26^ The basically consistent maturity and similar kerogen type exert a small influence on TOC for study samples.

### 5.2 Redox Conditions and Paleosalinity

**Figure 8:**
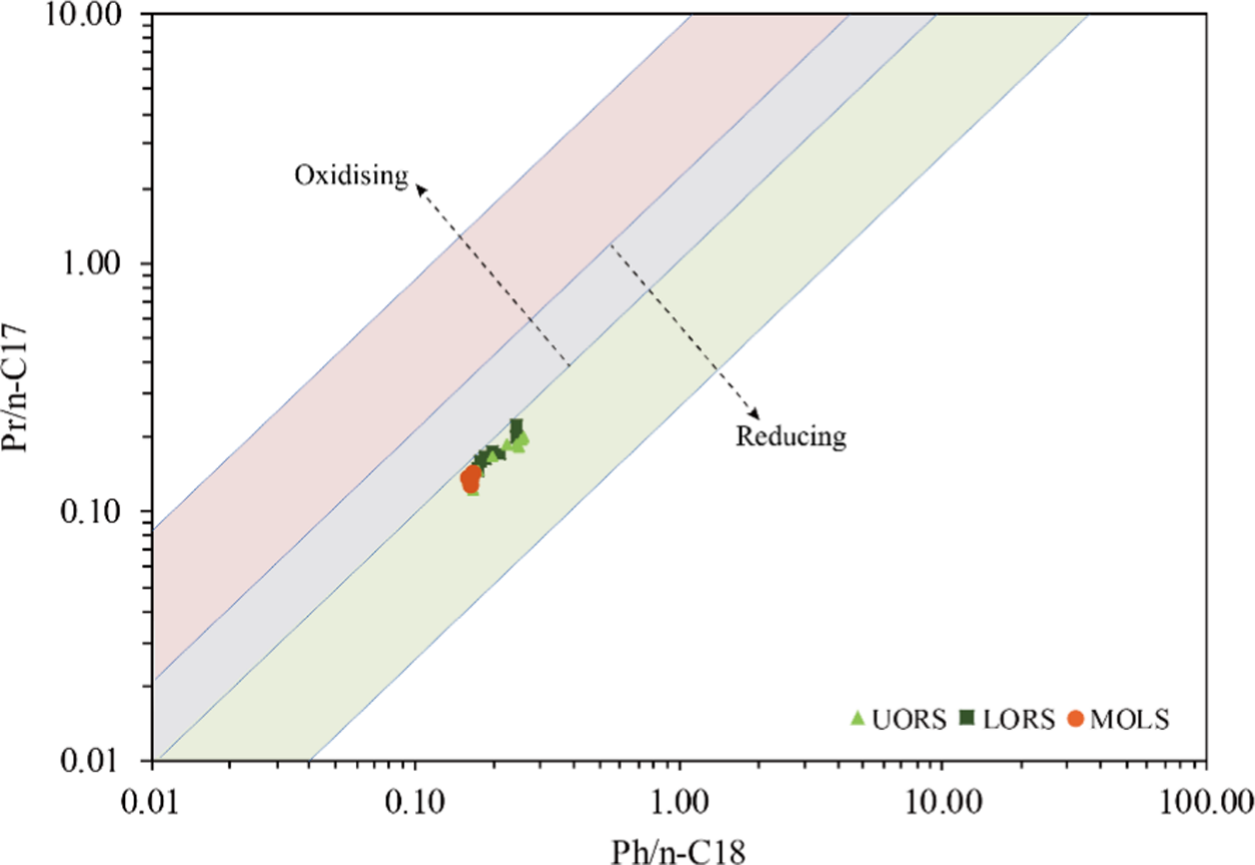
Pr/*n*-C_17_ versus Ph/*n*-C_18_ ratios for the Chang7_3_ samples.

The Pr/Ph ratio is commonly used to indicate the redox conditions of the depositional environment. A Pr/Ph ratio of >3.0 often implies an oxic condition, which is accompanied by the input of terrestrial organic matter, while the ratio of <0.8 generally indicates an anoxic depositional condition.^27^ The Pr/Ph ratio values in the studied samples vary in a narrow range (mainly 0.7–0.9), indicating that the Chang7_3_ submember was deposited in a weak-reducing condition. The plot of Pr/*n*-C_17_ versus Ph/*n*-C_18_ (Figure 8) also suggests the weakly reducing depositional environment of the Chang7_3_ submember. It should be noted that these parameters can also be affected by the maturity of organic matter, however, for samples of the present study having a basically consistent maturity make these parameters reliable to predict the depositional environment.

**Figure 9:**
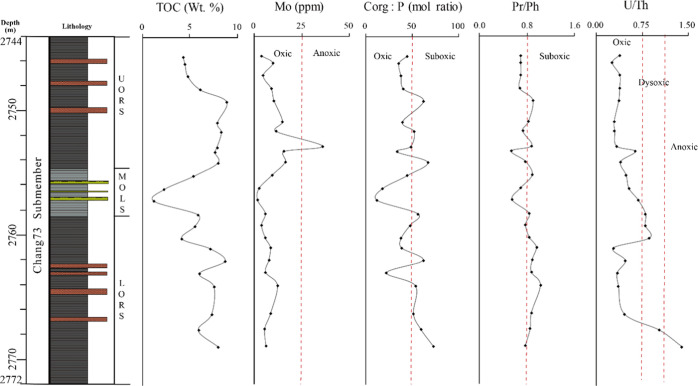
Profiles of redox proxies for the Chang7_3_ submember of Well F75.

Bottom-water oxygen concentration plays a great role in retaining phosphorus (P) within sediments, with the oxygenated bottom-water environment promoting P retention in sediments but the anoxic environment strengthening the removal of P from sediments to the overlying water column.^28^ Algeo^29^ further proposed the *C*_org_/*P* ratio to indicate different redox conditions, with the *C*_org_/*P* ratio commonly <50 for oxic conditions, 50–100 for suboxic conditions, and >100 for anoxic conditions. The *C*_org_/*P* ratio values in the investigated samples are in the range of 14–69, suggesting an oxic to suboxic setting (Figure 9). It should be noted that the reliability of the *C*_org_/*P* ratio can be affected by the age of sediments and the history of sediments.^30^ According to Jones,^31^ compared with other redox-sensitive trace-element ratios, such as Ni/Co and V/(V + Ni), the result of U/Th is a more reliable depositional condition indicator and the U/Th ratio < 0.75 suggests oxic conditions, 0.75–1.25 dysoxic, and >1.25 suboxic–anoxic. The U/Th values of studied samples vary from 0.24 to 1.4, with an average value of 0.53 (Table 3), indicating an oxic to suboxic redox condition during the deposition of the Chang7_3_ shales (Figure 9). The abundance of molybdenum (Mo) in sediments is highly related to the organic richness, and its concentration of >25 ppm is generally indicative of deposition under anoxic and euxinic environments.^32^ The Mo contents are markedly lower than 25 ppm in the studied samples except for one elevated value of 36 ppm in the UORS (Figure 9). It should be noted that enrichment in these trace elements can be markedly different below or above a certain TOC threshold value.^33^ Collectively, these redox proxies reflect that weak-reducing to suboxic conditions prevailed with limited occurrence of anoxic and euxinic conditions during deposition of study sections.

**Figure 10:**
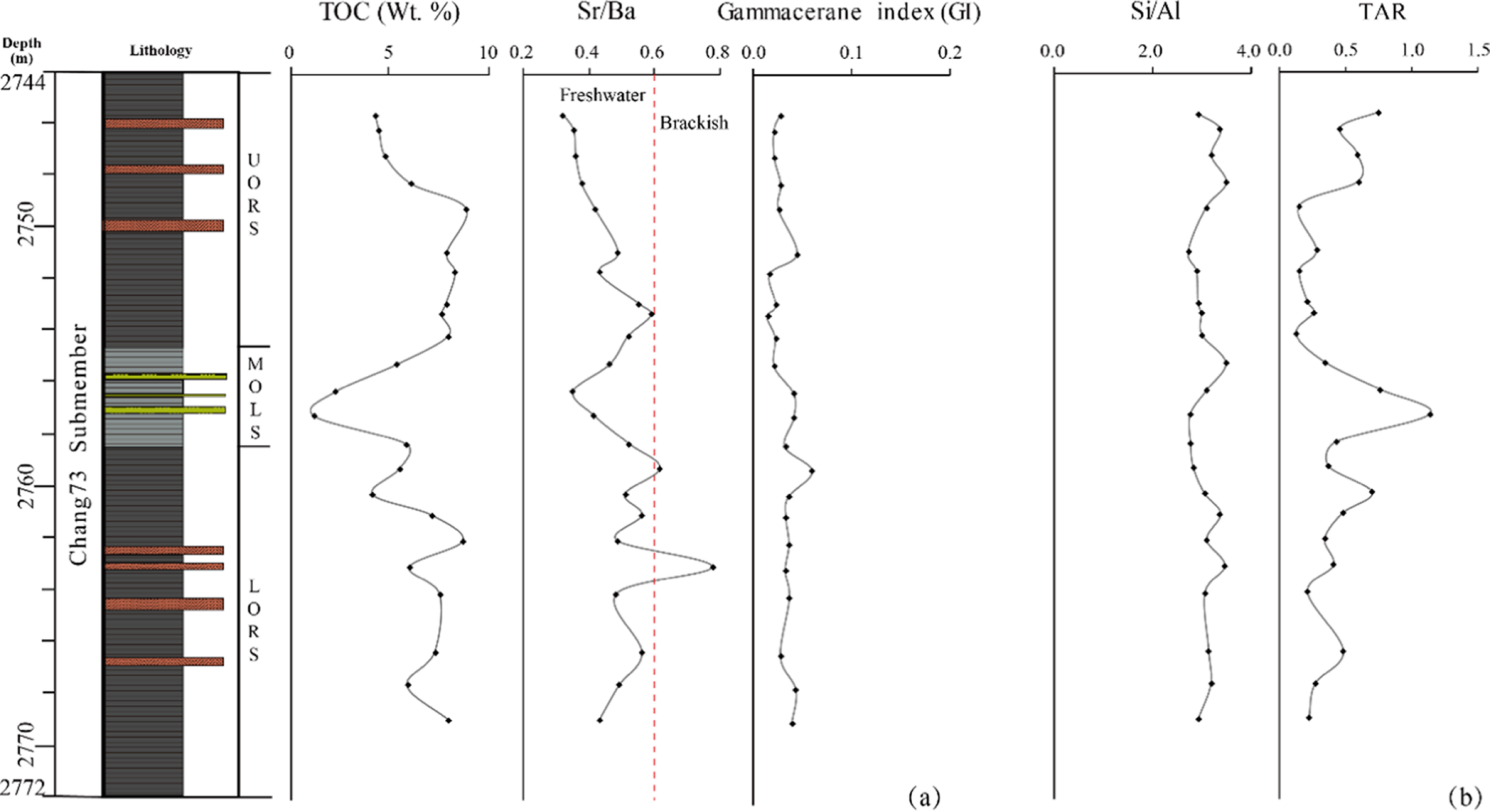
Profiles of (a) paleosalinity proxies and (b) terrestrial sediment input proxies for the Chang7_3_ submember of Well F75.

The high abundance values of gammacerane and the corresponding gammacerane index (GI = gammacerane/17α(H), 21β(H) C_30_ hopane) usually serve as indicators of saline-hypersaline depositional environments and waterbody stratification.^34,35^ The presence of low-abundance gammacerane and very low GI values (0.01–0.06, average 0.03) imply no development of saline water settings and waterbody stratification during the deposition of the Chang7_3_ submember. The ratio of Sr/Ba can be used as an indicator of water salinity, with Sr/Ba < 0.6 signifying freshwater; 0.6 < Sr/Ba < 1.0, brackish water; and Sr/Ba > 1.0, saline water.^36,37^ However, it should be noted that this parameter is not suitable for samples with a high concentration of CaO, which benefits the substitution of Sr^2+^ for Ca^2+^ and leads to unreliable Sr/Ba ratios.^38^ A relatively low concentration of CaO (mostly less than 2.0%) in studied samples and Sr/Ba ratio values of generally <0.6 (Table 3 and Figure 10a) indicate a fresh-brackish water environment for the study sections. The distribution feature of homohopanes (C_31_H > C_32_H > C_33_H > C_34_H > C_35_H) from all samples further suggests freshwater depositional environments (Figure 6). Collectively, these parameters indicate that UORS, MOLS, and LORS were developed dominantly in freshwater environments with brackish settings developing within a certain duration of the LORS.

### 5.3 Dilution

A large influx of terrestrial sediments can dilute the richness of organic matter by affecting the depositional rate or reducing the proportion of organic matter relative to the inorganic matrix.^5,39^ Higher TAR values (Figure 10b) and distribution characters (Figure 5) of the *n*-alkanes in the MOLS indicate an increased input of terrestrial organic matter into the paleolake. Increased levels of terrestrial organic matter may reflect the overall rise in terrestrial influx, which can result in dilution of organic matter richness within the MOLS, as evidenced by the apparently lower TOC contents compared to the UORS and LORS. However, Si/Al, an indicator of detrital influx,^40,41^ shows constant variation across the UORS, MOLS, and LORS (Figure 10b), implying that no pronounced terrestrial sediment dilution effect happened during the deposition of the studied sections.

**Figure 11:**
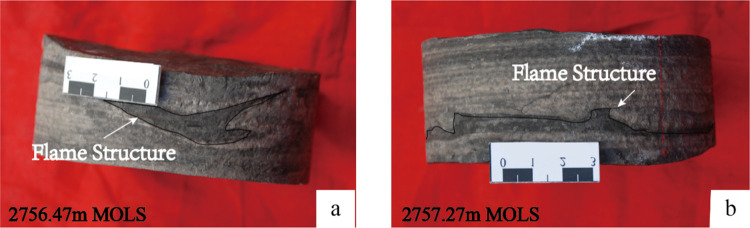
Core photo of the flame structure in Well F75: (a) 2756.47 m and (b) 2757.27 m.

Dark rock color, multiple rhythmic layers of siltstone and mudstone, and flame structure (Figure 11) indicate turbidite deposition within a certain interval of MOLS. The high energy of the coarse clastic terrestrial input eroded the underlying muddy sediment and formed flame structures (Figure 11). According to investigation of petrography, sedimentation, and field observations, previous studies^42,43^ attributed the coarse clastic terrestrial sediments within the Chang7_3_ submember close to the study area to turbidity currents or gravity flows.

Additionally, according to Müller,^44^ a 10-fold increase in the deposition rate will result in a 2-fold increase of TOC contents in sediments, provided that other factors affecting the abundance of organic matter remain about constant. Although both UORS and LORS show approximately 2-fold TOC content compared with that in MOLS, the deposition rate in the same location with a similar lithology seldom differs 10-fold,^44^ indicating that other factors, such as paleoproductivity and redox conditions, play major controls on the organic matter enrichment.

### 5.4 Paleoproductivity

Phosphorus, consisting of organic phosphorus (*P*_org_) and inorganic phosphorus (detrital P),^29^ is an indispensable nutrient for all organic organisms.^45^ The larger the influx of *P*_org_, the higher the paleoproductivity is.^46^ The *P*_org_ contents of samples calculated from the total P by subtracting a detrital phosphorus fraction (*P*_detr_) can be estimated from each sample’s Al content as shown in the following equation proposed by Schoepfer^46^
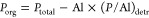
where (P/Al)_detr_ is equivalent to 0.0087 based on the average P and Al concentrations of the upper continental crust.^47^

**Figure 12:**
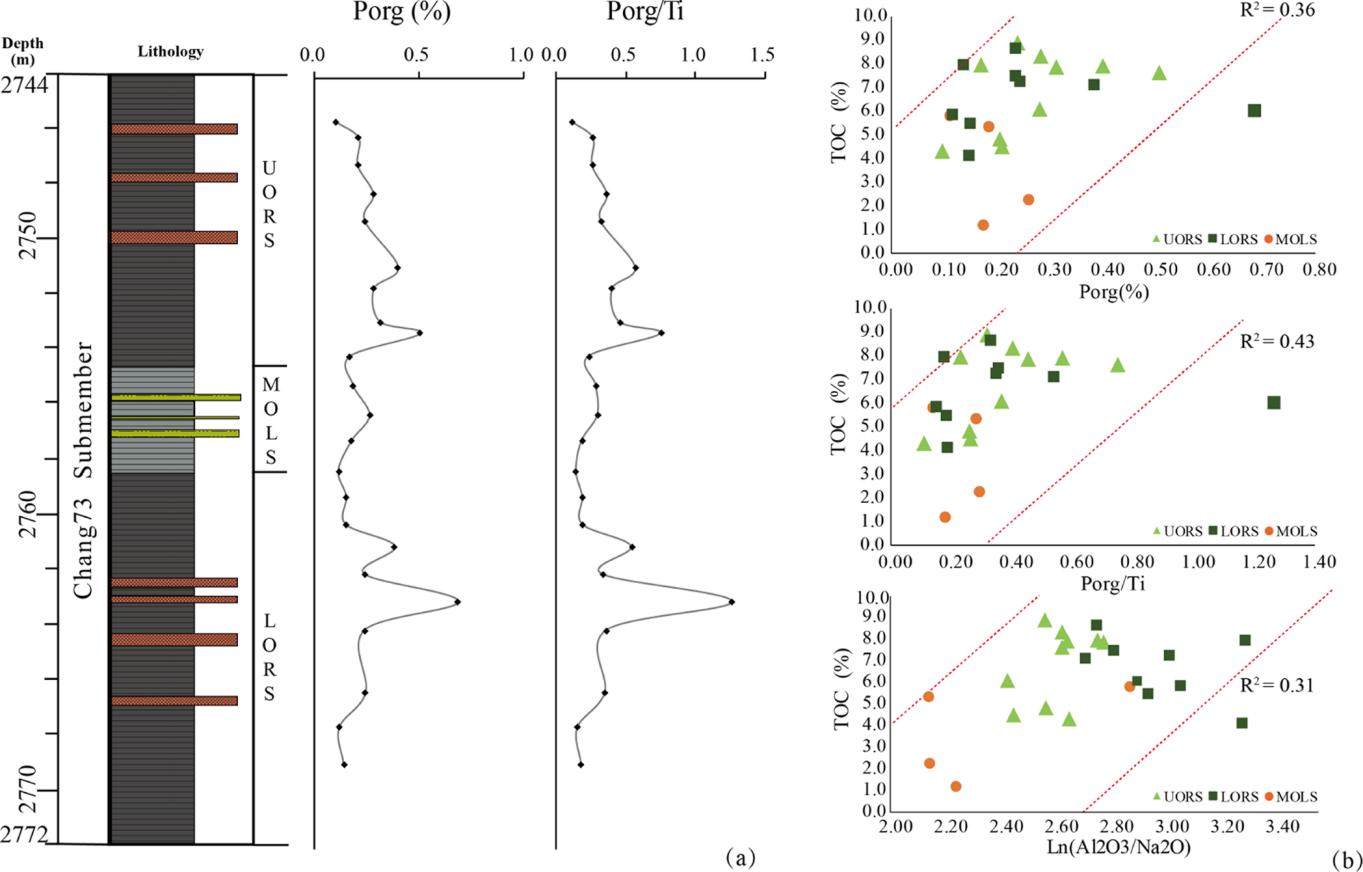
(a) Profiles of paleoproductivity proxies for the Chang7_3_ submember of Well F75; (b) cross-plot of TOC versus *P*_org_ (%), *P*_org_/Ti, and Ln (Al_2_O_3_/Na_2_O) for the Chang7_3_ submember of Well F75.

Samples in UORS and LORS have higher *P*_org_ contents (average 0.27 and 0.26%, respectively) than those within the MOLS (average 0.18%) (Table 3 and Figure 12a), indicating lower paleoproductivity during the deposition of MOLS. By excluding the influence of terrigenous clastic materials, the P/Ti ratio was used as an indicator of paleoproductivity.^33^ Thus, the *P*_org_/Ti ratio was used to evaluate paleoproductivity in the studied sections. Samples in the MOLS show lower *P*_org_/Ti ratios than those in the UORS and LORS (Figure 12a), implying obvious paleoproductivity fluctuations during deposition of the study sections with higher paleoproductivity in the UORS and LORS than that in the MOLS. Aquatic phytoplanktons are usually recognized as the origin of C_27_ steranes,^48^ while C_29_ steranes are commonly derived from terrestrial plants.^49^ The L- and V-type sterane distributions of most samples from the Chang7_3_ (Figure 6) indicate that aquatic organisms make a major contribution to the organic matter accumulated in the sections and thus play a dominant role in the fluctuating paleoproductivity.

### 5.5 Paleoclimate

**Figure 13:**
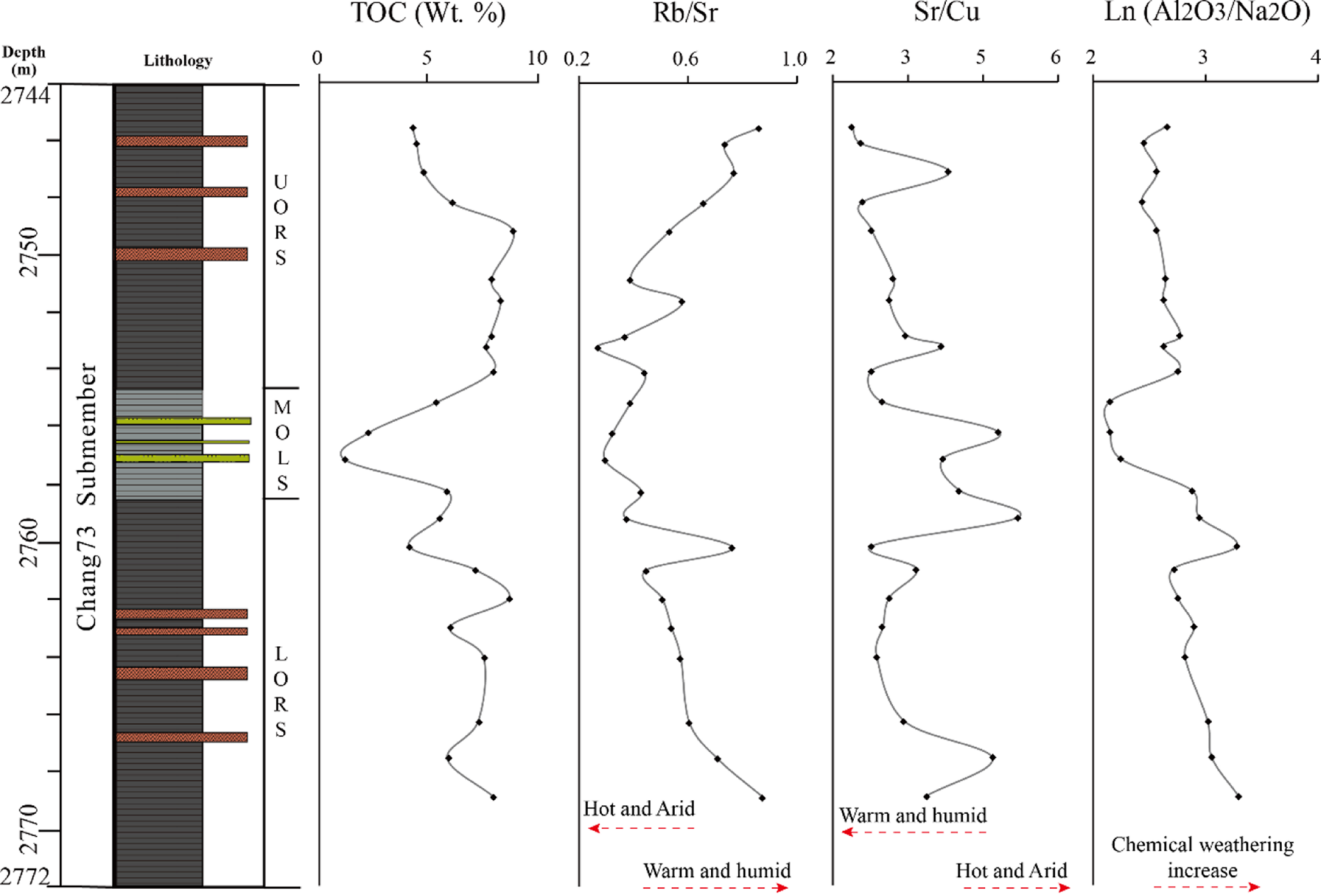
Profiles of paleoclimate proxies and chemical weathering proxy for the Chang7_3_ submember of Well F75.

The Sr/Cu ratio commonly serves as a proxy of paleoclimate.^50,51^ Arid climate is beneficial for enriching Sr, whereas Cu is usually concentrated under humid conditions.^52^ The lower the Sr/Cu ratio is, the warmer and more humid the paleoclimate would be. However, the ratios of Rb/Sr imply quite contrasting paleoclimate conditions compared with Sr/Cu ratios.^53^ Noticeably elevated Sr/Cu ratios and decreased Rb/Sr ratios within the MOLS (Figure 13) indicate a hot and dry paleoclimate, which is quite different from the warm and humid paleoclimate in the UORS and LORS, as indicated by the relatively low Sr/Cu and high Rb/Sr ratios. Under warm and humid climate conditions, precipitation will increase and further strengthen chemical weathering. Ln(Al_2_O_3_/Na_2_O), a better indicator to reflect changes of the chemical weathering degree than the chemical index of alteration (CIA), shows high values when the degree of chemical weathering elevated which commonly caused by heavy rainfall.^54,55^ Higher values of Ln(Al_2_O_3_/Na_2_O) within the UORS and LORS (Figure 13) indicate much stronger chemical weathering conditions than the MOLS, thus further indicating a warmer and more humid paleoclimate during the deposition of the UORS and LORS compared to the MOLS, which was deposited under a hot and arid paleoclimate.

### 5.6 Volcanic Eruption

Multiple tuff intervals were observed in the Chang7_3_ submember (Figure 2), suggesting frequent volcanic eruptions during the corresponding period. Previous studies^19,56^ have illustrated that volcanic events happened in the Late Triassic Chang7 deposition period, dominantly intermediate-acidic Plinian types, and are consistent with the distribution of the Chang7 organic-matter-rich shales both in time and in space. Explosive volcanic eruptions can produce great amounts of volcanic-ash-containing metal salts.^57,58^ Long-range transport of volcanic ash through air convection and final deposition into aqueous environments may lead to the dissolution of absorbed metal salts and aerosols, providing key nutrients for organisms and increasing the primary bioproductivity,^59,60^ which can be termed as volcanic fertilization.

The average contents of major elements of the tuff and black shales within the Chang7_3_ submember are shown in Table 4, and data of fresh intermediate-acidic volcanic dust samples were also collected from the literature.^61−65^

**Figure 14:**
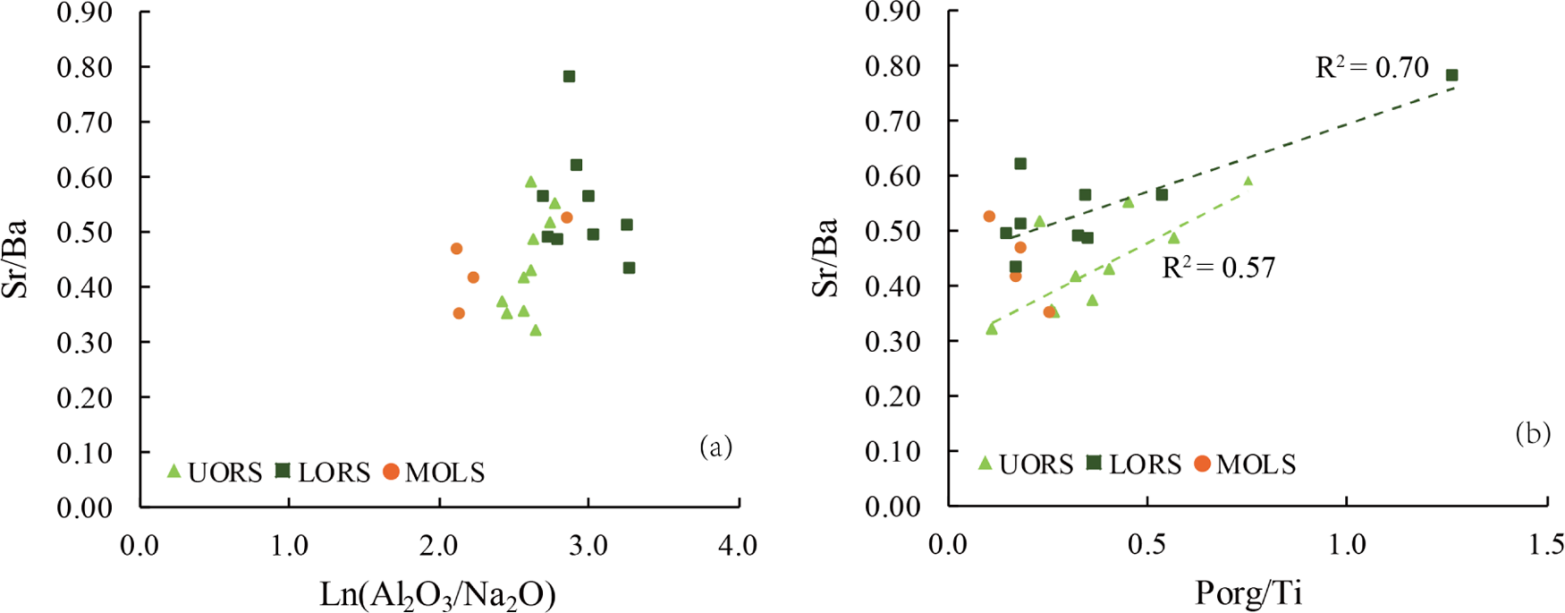
Plots of (a) Sr/Ba versus Ln (Al_2_O_3_/Na_2_O) and (b) Sr/Ba versus *P*_org_/Ti for the Chang7_3_ submember of Well F75.

The Chang7_3_ organic-rich shales contain a notably high abundance of nutrient contents such as P_2_O_5_ and Fe compared with fresh volcanic dust (Table 4) and much higher than those of Chang7_3_ tuff, which indicates that nutrients were transported and concentrated in the neighboring shales after deposition and hydrolyzation of volcanic ashes. The no obvious positive correlation (Figure 14a) between Sr/Ba and Ln(Al_2_O_3_/Na_2_O) may indicate that the increased lake-water salinity was due to input of volcanic ash, which is usually covered with soluble salt coatings.^65^ The significantly positive correlation between Sr/Ba and *P*_org_/Ti in UORS (*R*^2^ = 0.57) and LORS (*R*^2^ = 0.70) (Figure 14b), with no apparent correlation within MOLS, further supports the argument that the flourishment of aquatic organisms partly benefited from the volcanic ash containing abundant nutrient salts. It has been revealed that modern volcanic fertilization in oceanic water commonly lasts for very short time periods.^60,66^ Although volcanic activities were frequent during the Chang7_3_, the period of volcanic-induced lake-water fertilization is like a flash compared with the geological time scale of million years. Thus, the volcanic eruptions should not be primarily responsible for organic matter accumulation in the Chang7_3_ submember.

### 5.7 Major Controls on Organic Accumulation

Basic factors including production, destruction, and dilution control organic enrichment in lacustrine sediments and other environments. Maximized production and minimized destruction and dilution can lead to optimum organic enrichment.^39^ Destruction of organic matter is mainly controlled by the content of oxygen supplied to lake waters. In poorly oxygenated water columns, microbial respiration was significantly constrained, thus preventing organic matter from being destroyed and favoring the preservation of organic matter.^67^ For study sections, the relatively low oxygen content characterized by chiefly weak-reducing to suboxic environments shows basically no obvious changes among the UORS, MOLS, and LORS. Additionally, seawater invasion and chemical stratification of lake water, both of which could enhance the preservation of organic matter by inhibiting renewing oxygen in bottom waters,^68^ did not happen during deposition of the Chang7_3_ submember as indicated by GI, Sr/Ba ratios, and homohopane distribution patterns. Preservation conditions of organic matter show no noteworthy differences and likely do not lead to such significant changes of organic matter abundance within the UORS, MOLS, and LORS. The organic matter within MOLS was diluted, to a certain extent, by influx of terrestrial sediments derived from turbidity currents and gravity flows as discussed above. Thus, the enrichment of organic matter within study sections is largely controlled by the paleoproductivity of the lake. Paleoproductivity proxies, *P*_org_/Ti and content of *P*_org_, indicate a higher paleoproductivity within the UORS and LORS compared to the MOLS and show a positive correlation with TOC (Figure 12b). Among many factors including wind, solar radiation input, precipitation, temperature, and water chemistry that could affect the overall paleoproductivity of organic matter in a lake, water chemistry and solar radiation input have the most significant impact.^69,70^ Water chemistry, which controls the availability of nutrients supporting organic matter production of a lake, is strongly influenced by basin hydrology and climate.^71^ A warm and humid climate is expected to increase precipitation and chemical weathering. Increased precipitation makes the river discharges increase and the lake level rise,^72^ which would trap clastic sediments near the shore and thus reduce clastic dilution.^73^ Enhanced precipitation in the catchment of the lake intensified chemical weathering and thus increased the delivery of soil-derived nutrients to the lake.^74^ During deposition of the UORS and LORS, fertile soils around the lake released a large quantity of nutrients into the deep lake developed under a warmer and more humid climate compared to the MOLS. Increased influx of nutrient loads resulted in more production of organic matter and might have triggered algal bloom in the waters. Tuff layers observed within the UORS and LORS imply multiple volcanic activities during deposition of the UORS and LORS. These nutrient-rich volcanic ash falling into the paleolake fueled aquatic plant growth and further increased paleoproductivity. Additionally, with the eruption of volcanos during the deposition of the UORS and LORS, a large amount of carbon dioxide released into the air and the partial pressure and availability of carbon dioxide increased, thereby promoting photosynthesis of aquatic plants within the lake and further supporting high paleoproductivity. The paleoclimate was relatively hot and dry during deposition of the MOLS, as indicated by Sr/Cu and Rb/Sr discussed above. The availability of nutrients that can be brought into the lake from the catchment area by surface runoffs decreased and made a great contribution to decreasing paleoproductivity during the MOLS. A relatively shallow lake developed within the MOLS due to reduction of precipitation under the stable subsidence background of the period.^75^ As a consequence of the lake level lowering, much more terrestrial organic matter input into the lake because of decreasing distance to the shoreline, where terrestrial organic matter preferentially accumulates^76^ leading to the change of kerogen types. It has been reported that monsoon activity played an important role in the accumulation of Chang7 black shales.^77^ It should be noted that frequent wind-induced resuspension of sediments in shallow lakes might also reduce paleoproductivity of the lake to a certain extent.

## 6 Conclusions

Extensive lacustrine organic-rich shales were deposited within the Chang7_3_ submember (divided into the UORS, MOLS, and LORS) in the Triassic Yanchang Formation of the Ordos Basin, North China. Organic matter dominated by type II kerogens in Well F75 currently falls in the main oil-generation window. Paleoredox conditions show no noteworthy variation, characterized by weakly reducing to suboxic conditions, across the three sections and favor preservation of accumulated organic matter. Factors that may lead to notable difference of organic matter abundance in study sections, including paleosalinity, clastic dilution, terrestrially derived nutrients, and paleoproductivity, were strongly controlled by the paleoclimate, which thus exerts a major impact on organic matter accumulation within the UORS, MOLS, and LORS.

Decreasing availability of terrestrially sourced nutrients under a hot and arid paleoclimate, along with increased clastic dilution, led to a lower paleoproductivity and lean organic matter accumulation within the MOLS. During deposition of the UORS and LORS, prevailed by a warm and humid paleoclimate, high influx of nutrients from continent lands, low clastic dilution, and high paleoproductivity boosted the enrichment of organic matter. Volcanic ash deposited during the UORS and LORS periods further promoted primary productivity of the lake by providing additional nutrient salts.
